# A Case Report on Breast Cancer-Related Lymphedema in Adulthood

**DOI:** 10.7759/cureus.25042

**Published:** 2022-05-16

**Authors:** Ruchika Kalra, Bhavna Anand, Harshita Sharma

**Affiliations:** 1 Physiotherapy, Amity Institute of Physiotherapy, Noida, IND; 2 Physiotherapy, Fortis Super Specialty Hospital, Delhi, IND

**Keywords:** mastectomy, complex decongestive therapy, physiotherapy, breast cancer related lymphedema, breast cancer

## Abstract

Breast cancer is a common occurrence in women, and it is also associated with lymphedema. In this case, the patient reported breast cancer-related lymphedema after a radical mastectomy with axillary lymph node dissection in the left breast; after her treatment, she experienced this condition for three months. Following the assessment, it was determined that the lymphedema was in stage 2 as a result of the delay. As the measurements increased, CDT was recommended for the treatment. This study provides evidence that early assessment and regular self-assessment for signs can lead to early resolution. With no cure, the only solution is to prevent and manage breast cancer lymphedema.

## Introduction

Breast cancer-related lymphedema is the most common complication arising after breast cancer surgery and treatment; a condition characterized by the presence of abnormal swelling in cases post-surgery or even years after treatment [1]. This condition cannot be cured, but it can be managed and avoided in the event of an occurrence [2]. According to statistics, one in every five women suffers from breast cancer-related lymphedema [3]. The etiology is surgery leading to the removal of lymph nodes, chemotherapy reducing lymph fluid circulation, and radiotherapy causing scarring of lymph nodes, which causes an imbalance in the lymphatic fluid, resulting in lymphedema in the affected breast and arm [4]. The physiotherapist evaluates and manages this condition using conservative and palliative care.

## Case presentation

**Figure 1:**
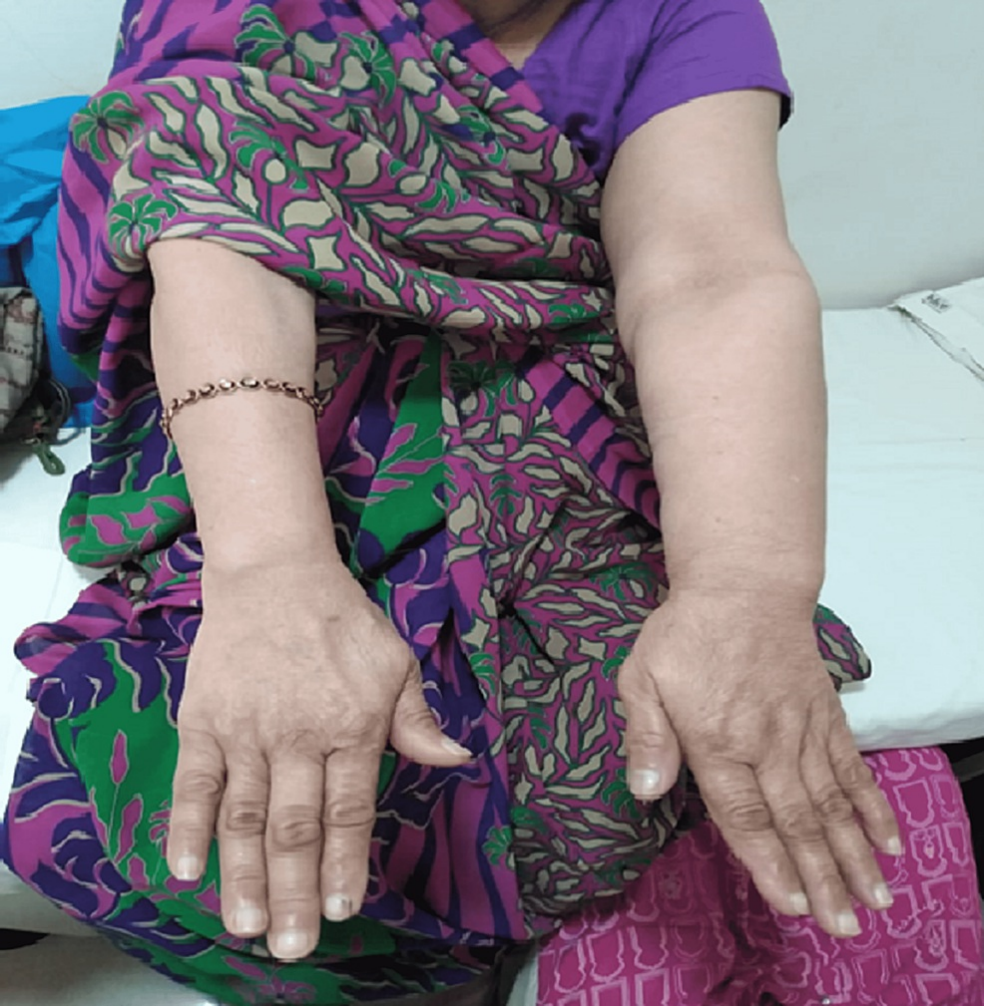
Breast cancer-related lymphedema in left upper limb

The patient was a 48-year-old female in the teaching profession, with three children who were breastfed, a family history of lung cancer, no history of smoking or drinking, and diabetes type 2 as a comorbidity. She experienced menopause at the age of 45. She was 48 years old when she felt a dull pain in her armpit and the inferolateral portion of her left breast, as well as tenderness in the nipple area. She went to see a gynaecologist, had a mammogram and was diagnosed with stage 2 breast cancer. She had a radical mastectomy and axillary lymph node dissection. She had 21 cycles of chemotherapy and four weeks of radiotherapy. She noticed heaviness and swelling in her left arm in the last three months after radiotherapy, but she didn't report it because of personal reasons (Figure 1). She went to see her onco-physiotherapist and was thoroughly evaluated. She was an endomorphic woman weighing 78 kg. The observation was providing an increase in limb size based on her condition. Her posture was observed, and she had developed a forward head neck. Her skin was firm, and a scar on the mastectomy line was visible. On palpation, she was found to have pitting oedema, a stage 2 lymphedema. The sensory evaluation was performed in accordance with the rules, but it turned out to be normal. She didn't have pain throughout her limbs, according to the pain assessment. The motor assessment revealed normal tone and a reduced range of motion in the affected arm in all planes (sagittal, frontal, and transverse) with a difference of approximately 15 degrees in active range of motion and 10 degrees in passive range of motion. Her muscle power in both upper limbs was measured using manual muscle testing, with the group of muscles targeted showing a decrease as a grade of 3 on the affected side and a grade of 4 on the non-affected side. She was circumferentially measured and her measurements increased when compared to the unaffected limb (Table 1).

**Table 1:** Circumferential measurements of both right and left upper limb

Point of measurement	Left arm	Right arm
Tip of shoulder	44 cm	41.0 cm
4 inches below shoulder tip	34.0 cm	33.0 cm
4 inches above elbow joint	31.3 cm	30.6 cm
At elbow joint	32.75 cm	28.8 cm
4 inches below elbow joint	31.8 cm	28.8 cm
4 inches above wrist joint	31.5 cm	29.32 cm
At the wrist joint	25.0 cm	21.2 cm
Palm to dorsum	22.3 cm	21.3 cm

The provisional diagnosis was breast cancer-related lymphedema in the left arm. Physiotherapeutic goals were established based on the problem list to reduce limb size, improve muscle strength, increase range of motion, and improve quality of life. A complex decongestive therapy plan was recommended, which included manual lymphatic drainage, exercises, and multi-layer bandaging (Table 2).

**Table 2:** Complex decongestive therapy protocol

Complex decongestive therapy	Protocol
Manual lymphatic drainage	40 minutes protocol: lymph node activation: for stretch and release of 10 cycles; manual lymphatic massage and manual lymphatic drainage: 10 circles each and drainage to active lymph nodes according to the division of arm and chest area
Exercises	For the left arm: 10 repetitions each exercise; wrist rotations; fanning of fingers; gripping of the wrist in flexion at 90, 180 and abduction of the shoulder at 90 degrees without stress ball; stretch for flexors and extensors of the wrist; shoulder rotations; shoulder shrugs; pectoralis stretches; deltoid stretch; triceps stretch; capsular stretch of shoulder
Multi-layer bandaging	For 20 hours: Finger bandaging covering from wrist to fingers to wrist; arm stockinette; foam layer in a tubular manner; three layers of bandaging with the first bandage from wrist to dorsum to palm to the wrist in a figure of eight to mid-forearm; second bandage from wrist to above in figure of eight; third bandage from where the first ended; fourth bandage from where the second ended to the tip of the shoulder in a figure of eight.

## Discussion

Breast cancer-related lymphedema is a complication of the disease. As a result of sedentary lifestyle adaptation after surgery and treatment side effects, the occurrence of lymphedema is quite high. Lymphedema is more than just oedema in the arm or chest; it can lead to axillary web syndrome, lymphosarcoma or infection due to lymph accumulation in the affected limb, permanent changes in the skin leading to fibrosis, neurological deficit affecting the majority of the quality of life in all concerns [5], including physical concerns such as inability to complete movement, feeling of discomfort, inactivity, and heaviness; in psychological concerns that result in embarrassment and a stifled social life; in practical concerns such as the inability to complete daily activities of living and making life easier functional [6]. This case study demonstrates that not only is cancer the problem to be solved, but that the focus should always be on the body changes that the body is requesting. Lymphedema is an easy task to resolve and manage if detected early, as delays are difficult and long-term to manage with the presence of some challenges [7].

Various studies report interventions such as manual lymphatic drainage, low-level laser therapy, intermittent pneumatic compression, multi-layer bandaging, compression bandages, Kinesio-taping and exercises. Out of which manual lymphatic drainage, exercises, and multi-layer bandaging together work as complex decongestive therapy. There are studies of various types of physiotherapeutic interventions with varying effects over a given time period. So far, complex decongestive therapy has established itself as the gold standard for lymphedema treatment [8]. Studies show that presenting with a greater amount of lymphedema, as seen in this case, is simple and quick to resolve, with progression possible with functional and strength training [9]. According to this study, there are various types of exercises provided in breast cancer-related lymphedema, aerobic training, gravity resisted training, and swimming. Yoga, resistance training, aqua lymph training not only improve function but also strengthen those muscle contractions which channel lymph fluid to be avoided in the future even after the problem has been resolved, out of which dynamic exercises in moderate and high-frequency intensities provided better results [10].

## Conclusions

This study reports a patient case report for breast cancer-related lymphedema, where she was stage zero post-treatment. Changes were slow and gradual as reported by the patient which she took lightly and didn’t report to the medical professional and no intervention was followed. After three months it led to stage 2, with the development of the heavy limb and higher circumferential measurements. This study provided the data assessment of the patient concluding that leniency can lead to severe issues, making the condition less manageable and negatively affecting the quality of life. 
